# Acute Gout Flare After Carboplatin/5‐Fluorouracil for Locally Advanced Head and Neck Squamous Cell Carcinoma

**DOI:** 10.1002/cnr2.70497

**Published:** 2026-02-20

**Authors:** Henry Zou, Anas Al Janadi, Zeyad Sako

**Affiliations:** ^1^ Michigan State University College of Human Medicine Grand Rapids Michigan USA; ^2^ Corewell Health Medical Oncology and Hematology Grand Rapids Michigan USA

**Keywords:** 5‐fluorouracil, carboplatin, gout, head and neck squamous cell carcinoma

## Abstract

**Background:**

Head and neck squamous cell carcinoma (HNSCC) is the sixth most common cancer globally. For locally advanced cases when upfront surgery or radiation are not feasible, a platinum agent (carboplatin or cisplatin) with 5‐fluorouracil (carbo‐5FU) and immunotherapy is the standard of care. We present the first documented case of acute gout flare in a HNSCC patient after initiation of carboplatin‐5‐fluorouracil.

**Case:**

A 67‐year‐old male patient with a remote history of gout and recurrent HPV‐associated oropharyngeal squamous cell carcinoma (OSCC) presented for evaluation and management of local recurrence. He underwent right tonsillectomy, chemoradiation, and naturopathic therapy over the past 9 years, but the OSCC recurred in the right neck. As the patient was deemed a poor surgical candidate and declined radiation therapy, he was counseled on treatment options and elected to proceed with palliative systemic carbo‐5FU therapy but experienced an acute gout flare after cycle 1. Uric acid was checked and found to be elevated at 9.3. He then started prednisone, allopurinol, and intravenous hydration for gout control, and his initially elevated uric acid levels normalized over the next 5 weeks.

**Conclusions:**

Our case describes the first documented incidence of gout flare precipitated by carbo‐5FU, but also demonstrates a successful pharmacological treatment approach to control chemotherapy‐induced gout.

## 1 Introduction

Head and neck squamous cell carcinoma (HNSCC) develops from mucosal squamous cells of the mouth, throat, and nasal cavity and is associated with tobacco use, excessive alcohol use, and human papillomavirus (HPV) [1]. It is the sixth most common cancer globally, causing 890 000 novel cases and 450 000 deaths in 2018, and its incidence rate is projected to rise by 30% by 2030. Platinum‐based chemotherapy (cisplatin or carboplatin) combined with 5‐fluorouracil (5FU) and immunotherapy regimen is the standard of care for metastatic, locally advanced or unresectable HNSCC [2, 3]. Carboplatin‐5FU (carbo‐5FU) activates proinflammatory cytokines, significantly raising the risk of toxic inflammatory adverse effects including sepsis, septic shock, nausea, and emesis [4]. Additionally, carbo‐5FU can induce metabolic adverse effects; carboplatin is associated with hyponatremia, hypokalemia, and dehydration, while 5FU is associated with hyperammonemic encephalopathy and hepatic steatosis [5, 6]. We present the first documented case of acute gout flare in a patient receiving carbo‐5FU for locally advanced unresectable HPV‐induced oropharyngeal squamous cell carcinoma (OSCC), a subtype of HNSCC.

## 2 Case

A 67‐year‐old male patient with a past medical history of chronic untreated gout and locally advanced, recurrent, HPV‐associated OSCC presented to Corewell Health Lemmon‐Holton Cancer Pavilion in January 2025 for evaluation and management of local recurrence of OSCC. His OSCC was initially diagnosed in July 2015 after he was evaluated for a right‐sided neck mass; diagnosis was confirmed via fine needle aspiration biopsy of two cervical lymph nodes (LNs). Computed tomography (CT) and positron emission tomography (PET) scans demonstrated a right‐sided necrotic level IIA nodal mass in the neck with mixed solid and cystic features and marked hypermetabolic activity in the right tonsillar pillar region. However, CT and PET were negative for mediastinal hilar or axillary lymphadenopathy or metastasis. He subsequently underwent a right tonsillectomy, which revealed an invasive, well‐differentiated, p16+ OSCC with keratinization focally positive deep margins on pathology.

In November 2015, a PET demonstrated two subcentimeter pulmonary lesions (laterality unspecified), and unilateral radiation therapy (RT) with concurrent cisplatin was recommended. Magnetic resonance imaging (MRI) in February 2016 after the PET showed a heterogeneously enhancing right level II neck mass 40 × 35 × 26 mm consistent with recurrence. However, the patient declined chemoradiation and instead established care at multiple oncology centers while undergoing 2 years of naturopathic therapy involving vitamin C infusions, intravenous (IV) salicinium, and cryotherapy. During these years, an MRI in July 2017 showed metastatic disease progression with level III adenopathy causing mass effect on the right internal jugular vein (IJV). Despite multiple urgent recommendations from various oncologists over these 2 years, the patient repeatedly deferred evidence‐based medical care in favor of naturopathic therapies.

**FIGURE 1:**
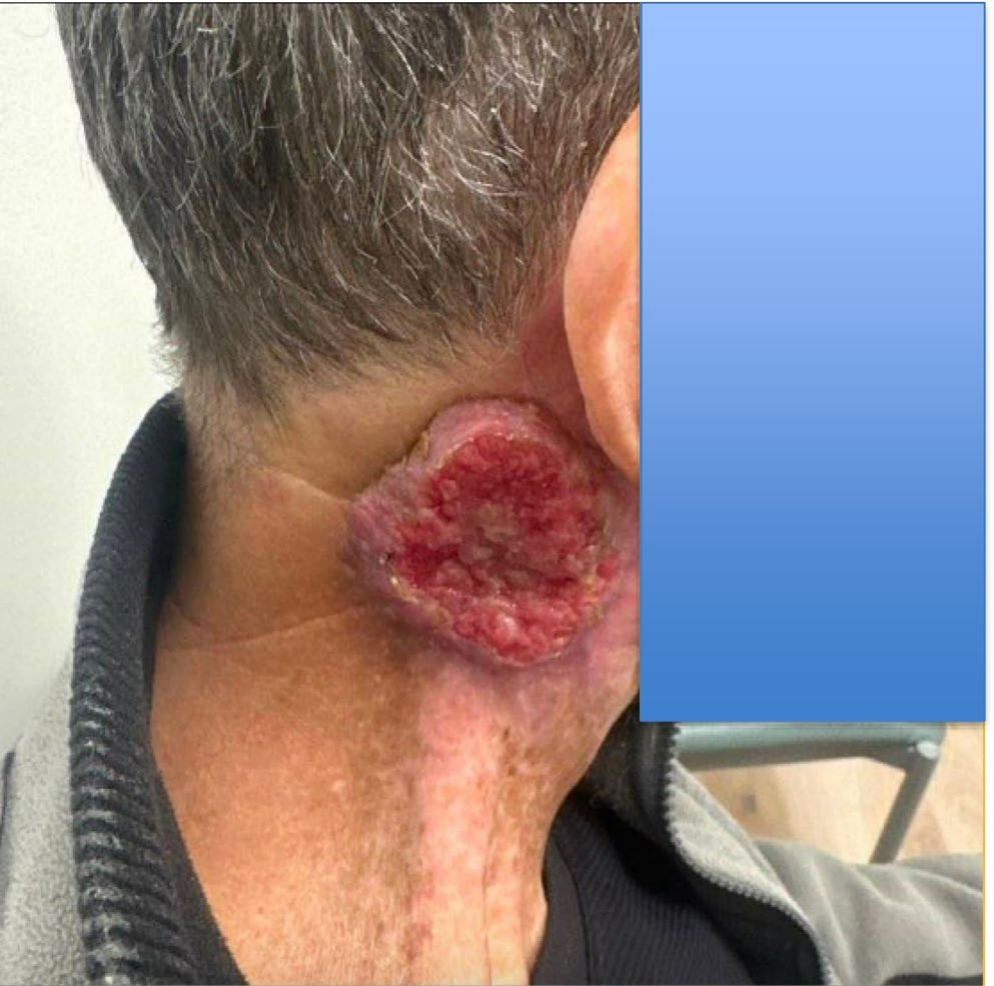
Markedly avid right neck OSCC with exophytic morphology extending to the skin surface in recurrence pattern.

After 2 years of disease progression on solely naturopathic therapy, the patient reported neck pain, voice changes, difficulty turning his head, and three areas of erythematous skin changes in April 2018. A PET in the same month showed massive right neck nodal involvement reaching 15 cm and significant cervical spine uptake. He subsequently completed docetaxel, cisplatin, and 5‐FU chemotherapy over 2 months (May to July) and concurrent RT over 5 months (May to September). Follow‐up CT in August 2018 showed interval decreased size of the right cervical nodal mass and supraclavicular LNs relative to the April PET and no intrathoracic metastatic disease. Surveillance laryngoscopy in June 2020 found no evidence of recurrent disease. However, in January 2025 the patient experienced recurrence with a markedly avid right neck malignancy that extended to the skin surface with exophytic morphology (Figure 1).

**FIGURE 2:**
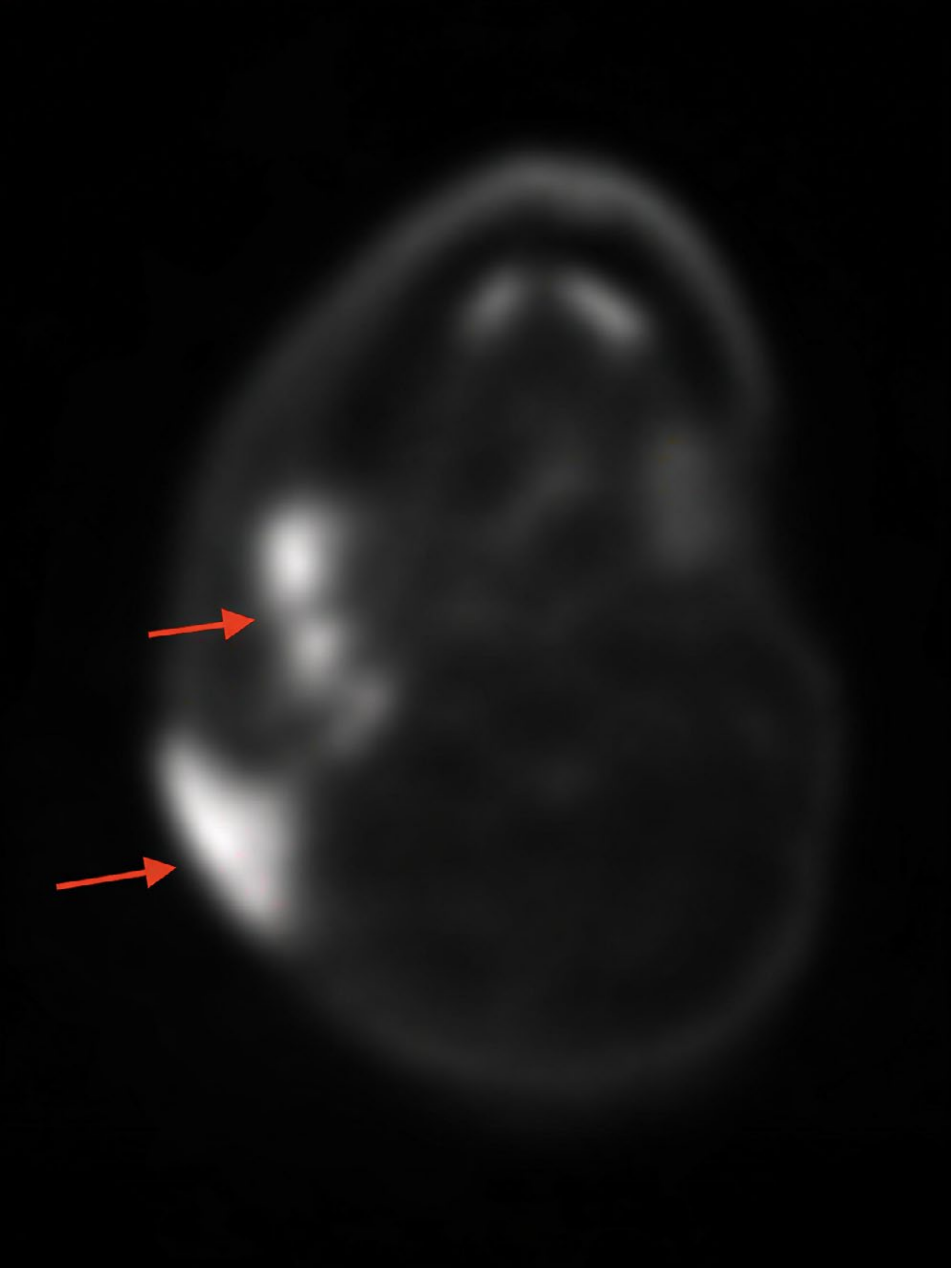
Neck PET/CT showing local soft tissue invasion and prominent right cervical lymph nodes with increased uptake concerning for metastasis (red arrows).

PET/CT showed local invasion of adjacent soft tissue structures without definite osseous invasion, occlusion of the right IJV, distortion of right neck anatomy, questionable right cervical LN metastases, and indeterminate left prominent cervical LNs with mild uptake (Figure 2).

The mediastinal and bilateral hilar LNs were prominent with uptake and borderline enlarged, but this was favored to reflect a systemic reactive process given the lack of superior mediastinal and inferior neck adenopathy and the diffusely mildly elevated bone marrow uptake.

**FIGURE 3:**
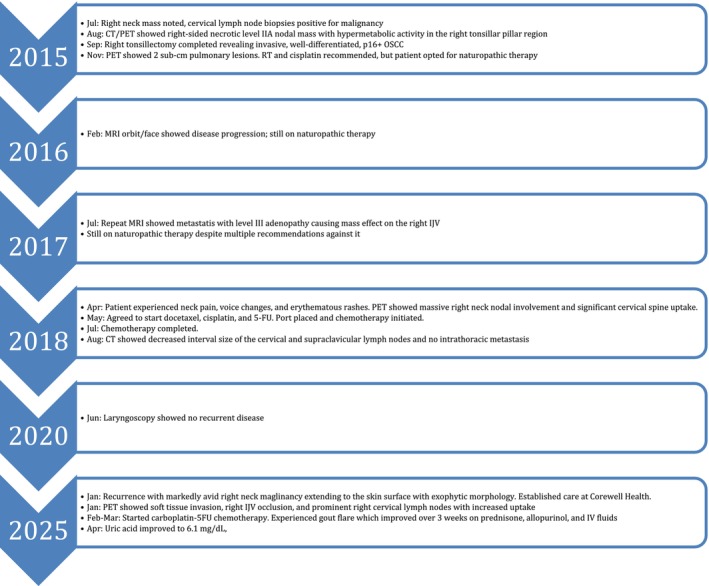
Timeline of disease course and management.

The patient was evaluated by surgical oncology but deemed a poor surgical candidate given the location of the malignancy; he also declined palliative RT. The patient was offered palliative systemic therapy, but declined immunotherapy over concerns of side effects despite being counseled on the potential efficacy of pembrolizumab given his 100% Programmed Death‐Ligand 1 Combined Positive Score. He instead started carboplatin‐5FU in February, but developed an acute gout flare after cycle 1 and was started on oral prednisone 40 mg daily and IV hydration as needed for gout control. The patient had an elevated uric acid of 9.3 mg/dL and experienced two gout flares over the past year despite dietary controls, so he was started on oral allopurinol 100 mg daily. After uric acid remained elevated at 9.5 mg/dL 1 week later in March, allopurinol was increased to 300 mg daily. After 3 weeks, the patient's uric acid decreased to 8.4 mg/dL and he completed cycle 2 without recurrence of gout flares, though he reported having intermittent neck pain and diarrhea. However, the right neck malignancy appeared stable on CT, though there was a spherical 8.5 mm enlarged right parotid LN. One week later in April, his uric acid decreased to 6.1 mg/dL, and the patient continues to be re‐evaluated with a Complete Metabolic Panel, Complete Blood Count, uric acid, magnesium, and office visit 3 weeks before starting further cycles (Figure 3).

## 3 Discussion

Carboplatin‐5FU is an accepted first line chemotherapy regimen for locally advanced and metastatic HNSCC; carboplatin is an alkylating agent that causes DNA strand cross‐linking, while 5‐fluorouracil is a thymidylate synthase inhibitor [2, 7, 8]. Relative to the cisplatin used in first‐line chemotherapy, carboplatin has lower ototoxicity, neurotoxicity, and gastrointestinal toxicity but higher bone marrow suppression [3].

Although rare, carboplatin has been associated with hyperuricemia due to nephrotoxicity, which can impair uric acid secretion and precipitate gout [9]. Furthermore, 5‐FU has been associated with increased serum uric acid and renal histopathologic changes in murine models [10]. However, allopurinol reduces 5‐fluorouracil toxicity by increasing the concentration of intracellular orotic acid, which competes with 5‐fluorouracil for orotate phosphoribosyl transferase and decreases the production of 5‐fluorouracil's cytotoxic metabolites [8]. There remains a lack of literature on acute gout associated with carbo‐5FU in solid tumor and HNSCC patients, which represented a significant challenge in this study. Our patient's post‐carbo‐5FU gout flare responded well to daily prednisone and allopurinol without recurrence thus far. Given that there have been no published cases of this adverse effect in HNSCC patients thus far, a significant finding of our study was the demonstration of a successful treatment model for this rare and previously undocumented reaction. Furthermore, it is surprising that our patient experienced an acute gout flare after carbo‐5FU, but not after cisplatin/docetaxel/5‐FU given that cisplatin demonstrates significantly higher rates of nephrotoxicity, which often impairs renal uric acid secretion [2]. However, while cisplatin is often associated with hyperuricemia due to proximal tubular toxicity, it can paradoxically cause hypouricemia due to renal wasting given uric acid's water solubility and small molecular weight [11].

Tumor lysis may represent an alternative explanation for our patient's acute gout flare. Tumor lysis syndrome (TLS) can precipitate hyperuricemia through the rapid release of purines from lysed tumor cells, though the most common associated complication is acute kidney injury due to the precipitation of uric acid crystals in renal tubules, not gout [12]. Our patient did not demonstrate the symptoms or electrolyte abnormalities characteristic of TLS. Nonetheless, milder complications of chemotherapy‐induced tumor lysis should be considered given our patient's significant tumor burden, possible undetected residual tumor following 2 years of disease progression on naturopathic therapy, and history of recurrent gout flares.

Gout is typically precipitated by chronic hyperuricemia in which monosodium urate (MSU) crystals progressively deposit in joints over time [13]. However, acute gout flare is precipitated by an innate immune response to MSU which activates the NLRP3 inflammasome in macrophages, leading to interleukin‐β release followed by neutrophil recruitment [13]. Carbo‐5FU also activates the innate immune system; carboplatin induces the release of damage‐associated molecular patterns, while 5‐FU activates the NLRP3 inflammasome in myeloid‐derived suppressor cells leading to interleukin‐1β release. These similar mechanisms of innate immune activation may explain how carbo‐5FU induced an acute gout flare in our patient, as he did not demonstrate clinical evidence of acute kidney injury or tumor lysis syndrome following carbo‐5FU initiation. As there is a lack of literature on acute gout flare after carbo‐5FU, there are currently no evidence‐based recommendations regarding preventive measures or contraindications for this adverse effect.

Finally, the patient's dietary habits and lifestyle may represent confounding factors when attempting to establish a correlation between carbo‐5FU and acute gout flare. Diets heavy in alcohol, particularly beer, fructose, and purines often found in meat/seafood, increase the risk of gout flares [13]. Chronic dehydration and obesity also increase the risk of gout [13]. While our patient is not obese, his long‐term diet and fluid intake may have contributed to his gout flares, particularly given his two prior episodes of gout flares in 2024 before he started chemotherapy.

## 4 Conclusions

Gout flare is an extremely rare adverse effect of carboplatin‐5FU and has not been documented in HNSCC patients. We present the first documented case of acute gout flare after carbo‐5FU in an OSCC patient which was successfully treated with oral prednisone and allopurinol. Our patient highlights the importance for clinicians to remain vigilant for this rare adverse effect and demonstrates a treatment approach to control chemotherapy‐induced gout.

## Author Contributions

**Henry Zou:** conceptualization, data curation, formal analysis, investigation, methodology, writing – original draft, visualization, validation, writing – review and editing. **Anas Al Janadi:** project administration, writing – review and editing. **Zeyad Sako:** conceptualization, methodology, project administration, writing – review and editing.

## Funding

The authors have nothing to report.

## Consent

The patient gave written informed consent for publication of case details and use of images.

## Conflicts of Interest

The authors declare no conflicts of interest.

## Data Availability

The data that support the findings of this study are available on request from the corresponding author. The data are not publicly available due to privacy or ethical restrictions.
